# MiRNA-Drug Resistance Association Prediction Through the Attentive Multimodal Graph Convolutional Network

**DOI:** 10.3389/fphar.2021.799108

**Published:** 2022-01-12

**Authors:** Yanqing Niu, Congzhi Song, Yuchong Gong, Wen Zhang

**Affiliations:** ^1^ School of Mathematics and Statistics, South-Central University for Nationalities, Wuhan, China; ^2^ College of Informatics, Huazhong Agricultural University, Wuhan, China; ^3^ School of Computer Science, Wuhan University, Wuhan, China

**Keywords:** miRNA-drug resistance association, graph convolutional network, multimodal, deep learning, attention neural network

## Abstract

MiRNAs can regulate genes encoding specific proteins which are related to the efficacy of drugs, and predicting miRNA-drug resistance associations is of great importance. In this work, we propose an attentive multimodal graph convolution network method (AMMGC) to predict miRNA-drug resistance associations. AMMGC learns the latent representations of drugs and miRNAs from four graph convolution sub-networks with distinctive combinations of features. Then, an attention neural network is employed to obtain attentive representations of drugs and miRNAs, and miRNA-drug resistance associations are predicted by the inner product of learned attentive representations. The computational experiments show that AMMGC outperforms other state-of-the-art methods and baseline methods, achieving the AUPR score of 0.2399 and the AUC score of 0.9467. The analysis demonstrates that leveraging multiple features of drugs and miRNAs can make a contribution to the miRNA-drug resistance association prediction. The usefulness of AMMGC is further validated by case studies.

## 1 Introduction

Drug development is an expensive and time-consuming process, and wet experiments are needed to select the drug targets and ensure the safety as well as the effectiveness of drugs. Although there are more than 25,000 protein-coding genes in the human genome, approved drugs can only target about 600 specific disease-related proteins (Hopkins and Groom, 2002; Overington et al., 2006). MicroRNAs (miRNAs) as one type of non-coding RNAs are identified as potential targets (Huang et al., 2019) due to their involvement in regulating the expression of related genes, and over-expressed/under-expressed expression of miRNAs could down-regulate/up-regulate genes with protein products necessary for drug efficacy/inhibiting drug function, and thus variations in miRNA profiling of patients is the cause of different therapeutics for individuals, especially the drug resistance. A thorough understanding of the impact of miRNA expression on drug resistance is important for drug discovery.

A few efforts have been made on predicting resistance associations between miRNAs and drugs. Dai et al. constructed the ncDR database (Dai et al., 2017), which contains comprehensive information about miRNA-drug resistance associations and laid a solid foundation for further computational analysis. By formulating known miRNA-drug resistance associations as a bipartite graph with miRNA and drug nodes, Huang et al. (Huang et al., 2019) proposed a graph convolution based miRNA-drug resistance association prediction model, named GCMDR, which uses miRNA expression profile and drug fingerprint as features. GCMDR produces satisfying results, but it still remains the space for improvement. A high-accuracy prediction model usually requires diverse information, which reflects different characteristics of drugs, miRNAs, and miRNA-drug resistance associations. Although miRNA expression profiles and drug substructures play important roles in the association prediction, more features about miRNAs and drugs should be taken into account to improve the performances. In recent years, the graph learning methods, especially graph neural networks (GNN), showed great success in biomedical association prediction (Mudiyanselage et al., 2020; Yu et al., 2020; Fu et al., 2021; Lei et al., 2021; Liu et al., 2021; Yang and Lei, 2021; Zhang et al., 2021a). Thus, it is necessary to develop GNN-based multimodal method to address above mentioned issues and improve the miRNA-drug resistance association prediction.

In this work, we propose a novel method, namely attentive multimodal graph convolutional network (AMMGC), to predict miRNA-drug resistance associations. We construct four graph convolution sub-networks that leverage distinctive combinations of features including drug fingerprints, drug label encoding, miRNA expression profiles, and miRNA GO-based similarities, and learn the latent representations of drugs and miRNAs from sub-networks independently. Then, an attention neural network is employed to obtain attentive representations of drugs and miRNAs from their respective latent representations. Finally, miRNA-drug resistance associations are predicted by the inner product of the attentive representations. The computational experiments show that AMMGC outperforms state-of-the-art and baseline methods. The experimental analyses reveal that leveraging multiple features of drugs and miRNAs can enhance the performance of the miRNA-drug resistance association prediction.

## 2 Materials

In this paper, we use a miRNA-drug resistance association dataset from the published work (Huang et al., 2019), which contains 3,338 miRNA-drug resistance associations between 754 miRNAs and 106 drugs. Moreover, we collect some side information of drugs and miRNAs to build our prediction model.

We consider two features of the drugs to give more insights into their characteristics. One is the PubChem substructure fingerprint (Wang et al., 2009), which represents drugs as 920-dimensional binary vectors. The other is the label/integer encoding of SMILES of drugs (Öztürk et al., 2018), in which each label of SMILES is represented by a integer (e.g., “*C*”:1, “*O*”:2, “*N*”:3, “ = ”:4, etc.). Considering the varied lengths of drug canonical SMILES, we set the dimensions of representations as 85, and then a drug is represented by an 85-dimensional vector. For the miRNAs, we consider two features to describe their biological information. One is the miRNA expression profile (Gillis et al., 2007), and the miRNA expression profile has 172 dimensions representing the expression levels of a single type of miRNAs in 172 different human tissues and cell lines. The other is the miRNA Gene Ontology (GO) functional similarity described in (Yang et al., 2018). The GO functional similarity has 2,587 dimensions, and each dimension denotes the similarity scores between the miRNA and other miRNAs concerning their gene regulation functions. Since the above features are not available for all miRNAs, we use the average of those miRNAs whose values are known to estimate the missing values of other miRNAs.

## 3 Methods

### 3.1 Problem Definition

Given *p* drugs, *q* miRNAs, and their resistance associations, our task is to predict novel miRNA-drug resistance associations by leveraging features of drugs and miRNAs as well as known associations. Formally, the associations between *p* drugs and *q* miRNAs can be formulated as an undirected graph, in which drugs and miRNAs are taken as nodes and their associations are taken as edges. The graph can be represented by a (*p* + *q*) × (*p* + *q*) adjacency matrix *A*, defined as $A=\left[\begin{array}{cc} 0 & B\\ B^{T} & 0 \end{array}\right]$ , where *B* is the association matrix in which each row represents a drug and each column represents a miRNA. If the *i*-th drug is associated with the *j*-th miRNA, *B*_*ij*_ = 1; otherwise, *B*_*ij*_ = 0.

### 3.2 Graph Convolutional Networks

Graph convolutional network (Kipf and Welling, 2017) employs convolution operation over graphs to learn node embeddings by using local graph structure and node features. Formally, given an undirected graph *G* with the node feature matrix *X* and the adjacency matrix *A*, a graph convolutional network updates embeddings of graph nodes with the following rule:

$$H_{l+1}=f\left({\tilde{D}}^{-\frac{1}{2}}\tilde{A}{\tilde{D}}^{-\frac{1}{2}}H_{l}W_{l}\right)$$ (1)

where $\tilde{A}=A+I_{n}$ is the adjacency matrix with added self connections of graph *G*, $\tilde{D}$ is the diagonal matrix such that ${\tilde{D}}_{ii}=\sum_{j=1}^{n}{\tilde{A}}_{ij}$ . For the *l*-th graph convolutional layer, *W*_*l*_ is a layer-specific trainable weight matrix, *H*_*l*_ is the matrix of node representations, and specifically *H*_0_ = *X*. *f* is an activation function, such as ReLU and Sigmoid.

### 3.3 Attentive Multimodal Graph Convolutional Network-Based Method

**FIGURE 1:**
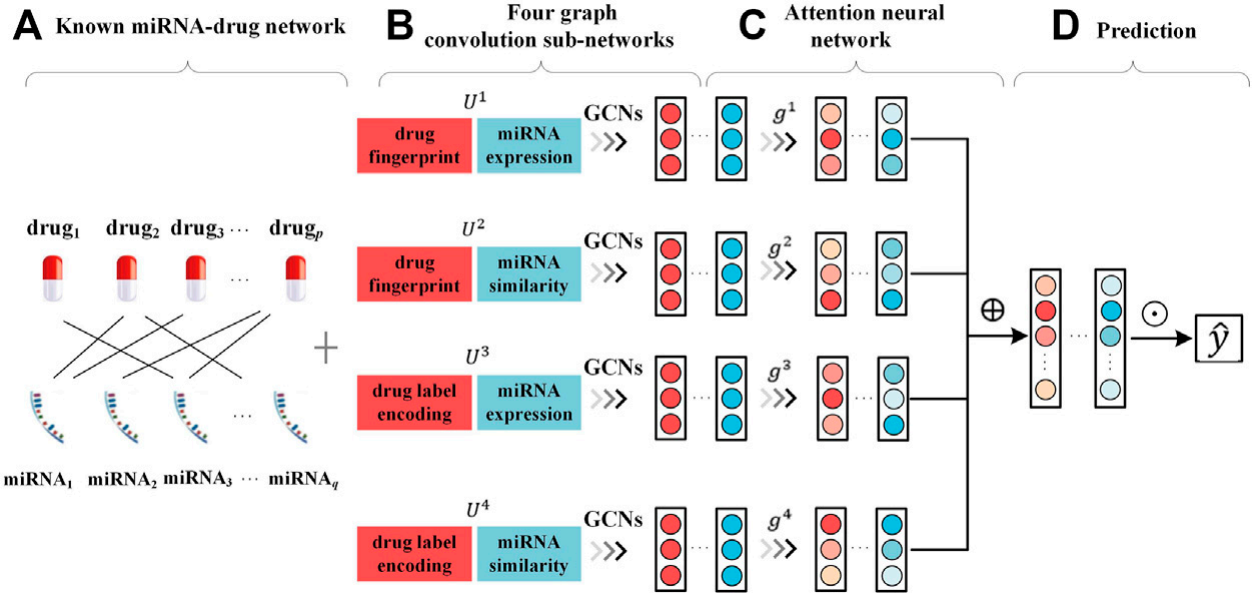
Framework of proposed method AMMGC **(A)**. Known miRNA-drug resistance association network. **(B)**. Learning node embeddings independently from four graph convolution sub-networks based on different feature combinations. **(C)**. Applying an attention neural network to learn attentive node embeddings. ⊕ denotes the concatenation operation. **(D)**. Using embeddings of drugs and miRNAs to produce the scores of miRNA-drug pairs. ⊙ denotes the inner product of drug embeddings and miRNA embeddings.

As shown in Figure 1, AMMGC constructs four graph convolution sub-networks with distinctive combinations of drug and miRNA features to obtain node embeddings from different views. After that, AMMGC applies an attention neural network to combine these node embeddings in different views for the miRNA-drug resistance association prediction.

As discussed in section 2, we have two drug features and two miRNA features, which are drug Pubchem fingerprint ( $X_{d1}\in R^{p\times 920}$ ), drug label encoding ( $X_{d2}\in R^{p\times 85}$ ), miRNA expression profile ( $X_{m1}\in R^{q\times 172}$ ), and miRNA GO-based functional similarity ( $X_{m2}\in R^{q\times 2587}$ ). AMMGC uses different combinations of drug features and miRNA features to build individual modals. Specifically, we consider four types of modals by using distinctive feature matrices as follows: *X* ^1^ = [*X*_*d*1_; *X*_*m*1_], *X* ^2^ = [*X*_*d*1_; *X*_*m*2_], *X* ^3^ = [*X*_*d*2_; *X*_*m*1_] and *X* ^4^ = [*X*_*d*2_; *X*_*m*2_]. Because the features of miRNAs and drugs have different dimensions, we add values of “0” to meet the same dimensions. For example, the dimension of drug fingerprint is 920 and the dimension of miRNA expression profile is 172, then we fill 748 values of “0” in front of the miRNA expression profile. For each individual modal *u*, we build a two-layer graph convolution sub-network. The input of each graph convolution sub-network are the feature matrix *X* ^*u*^ ∈ {*X* ^1^, *X* ^2^, *X* ^3^, *X* ^4^} and the known miRNA-drug resistance graph with adjacent matrix *A*, and the aggregation operation is formulated as:

$$E^{u}=ReLU\left({\tilde{D}}^{-\frac{1}{2}}\tilde{A}{\tilde{D}}^{-\frac{1}{2}}\left(ReLU\left({\tilde{D}}^{-\frac{1}{2}}\tilde{A}{\tilde{D}}^{-\frac{1}{2}}X^{u}W_{0}\right)\right)W_{1}\right)$$ (2)

where *W*_0_ and *W*_1_ are weight matrices of two GCN layers, $E^{u}\in R^{\left(p+q\right)\times d}$ is the embeddings of drugs and miRNAs in modal *u*, and *d* is the dimension of the embeddings. The prediction score between *i*-th drug and *j*-th miRNA in modal *u* is calculated by the inner product as follows:

$${\hat{y}}^{u}=Sigmoid\left(E_{i}^{u}{E_{p+j}^{u}}^{T}\right)$$ (3)

where $E_{i}^{u}$ and $E_{p+j}^{u}$ denote the *i*-th drug embedding and the *j*-th miRNA embedding in modal *u*, respectively.

Graph convolution sub-networks contain different types of information, and the node embeddings in a specific modal can only reflect information from one aspect. It is necessary to fuse multiple node embeddings in different views. It is assumed that embeddings in different modals are not equally contributed to the associations, and we employ an attention neural network to learn the weight of multiple node embeddings. For each modal *u*, we first project the original embedding matrix to an unnormalized matrix *g* ^*u*^ = *W*_*u*_ *E* ^*u*^ + *b*_*u*_. To reduce the computational complexity, we let *g* ^*u*^ to be a vector, and form the weighted embedding matrix by $E^{u}*\mathrm{diag}\left(g^{u}\right)$ . In this way, the size of parameter *W*_*u*_ is reduced from (*p* + *q*) × (*p* + *q*) to 1 × (*p* + *q*), and *b*_*u*_ is reduced from (*p* + *q*) × *d* to 1 × *d*. The learned attentive embedding matrix *Z* is calculated by:

$$Z={\|}_{u=1}^{4}\left(E^{u}*\mathrm{diag}\left(g^{u}\right)\right)$$ (4)

The prediction score $\hat{y}$ between *i*-th drug and *j*-th miRNA is calculated by:

$$\hat{y}=Sigmoid\left(Z_{i}{Z_{p+j}}^{T}\right)$$ (5)

where *Z*_*i*_ and *Z*_*p*+*j*_ denote the *i*-th attentive drug embedding the *j*-th attentive miRNA embedding.

### 3.4 Model Training

In the model training with two steps, the node embeddings are learned from sub-networks and then are served as the input of the attention neural network to make predictions.

The graph convolution sub-network for each modal *u* is trained independently, and we use the binary cross-entropy loss function formulated as:

$$L_{1}=-\overset{N}{\underset{s=1}{\sum }}\left(y_{s}\log\left({\hat{y}}_{s}^{u}\right)+\left(1-y_{s}\right)\log\left(1-{\hat{y}}_{s}^{u}\right)\right)$$ (6)

where *N* denotes the number of samples (miRNA-drug pairs), *y*_*s*_ is the true label of *s*-th sample and ${\hat{y}}_{s}^{u}$ is the prediction score of *s*-th sample of modal *u*.

For the attention neural network, we adopt the weighted binary cross-entropy classification loss function as:

$$L_{2}=-\overset{N}{\underset{s=1}{\sum }}\left(y_{s}\log\left({\hat{y}}_{s}\right)\times pos\text{\_}weight+\left(1-y_{s}\right)\log\left(1-{\hat{y}}_{s}\right)\right)$$ (7)

where ${\hat{y}}_{s}$ is the prediction score of *s*-th sample, *pos*_*weight* is a fixed scalar, and we set *pos*_*weight* equal to (*p* × *q* − *N*)/*N*. The *pos*_*weight* is used to balance the ratio between positive samples and negative samples.

We minimize the loss *L*_1_ and *L*_2_ using Adam optimizer (Abadi et al., 2016), and set the learning rate equal to 0.01. In sub-networks, we set the hidden units of two GCN layers to 128 and 256. More details of parameter settings can be seen in section 4.4.

## 4 Experiments

### 4.1 Experimental Setting

We adopt 5-fold cross-validation (5-CV) to evaluate the performance of AMMGC. The known miRNA-drug resistance associations are randomly equally divided into five subsets. In each fold, one subset of known associations is used for testing, and the remaining four subsets of associations are used for training. Specifically, we use four subsets of association pairs as positive instances and randomly select an equal number of samples from other pairs as negative instances to train the prediction model. We adopt the following evaluation metrics: the area under the precise-recall curve (AUPR), the area under the receiver-operating characteristic curve (AUC), F1-measure (F1), accuracy (ACC), and recall (REC). To avoid the bias of data split, we implement 10 runs of 5-fold cross-validation for each model, and the average metric scores and standard deviations are calculated.

### 4.2 Performance Comparison

We compare our method with four baselines and one state-of-the-art miRNA-drug resistance association prediction method:

• **Collaborative filtering(CF)**: Collaborative filtering (Su and Khoshgoftaar, 2009) is a classical recommendation algorithm.
• **Label propagation(LP)**: We consider the LP model mentioned in (Zhang et al., 2021b) as a baseline. The LP method propagates the existing miRNA-drug association information in the network to predict new associations.
• **Graph factorization(GF)**: Graph factorization (Ahmed et al., 2013) is a factorization-based network embedding method.
• **Structural Deep Network Embedding (SDNE)**: SDNE (Wang et al., 2016) is a deep learning-based network embedding method.
• **GCMDR**: GCMDR (Huang et al., 2019) makes use of graph convolution to build a latent factor model, which utilizes miRNA expression profile and drug fingerprint.

The 5-CV performances of all prediction models are shown in Table 1. In general, the proposed method AMMGC significantly outperforms four baselines in terms of most metrics. Specifically, AMMGC achieves an AUC of 0.946 7, which is almost 7.7*%* higher than that of the four baselines. This result shows that combining multiple features of drugs and miRNAs benefits the prediction of miRNA-drug pairs more than only using known miRNA-drug associations. Moreover, AMMGC also performs almost 4.89*%* higher than GCMDR on REC metric which is important for the study, verifying the efficacy and superiority of AMMGC. The substantial improvement of AMMGC over these compared methods is mainly attributed to two aspects: 1) AMMGC leverages the advantages of multiple features and applies them to build multimodal graph convolution sub-networks, while GCMDR only considers drug fingerprint and miRNA expression profile to build the prediction model; 2) AMMGC considers different contributions of embeddings learned from multimodal graph convolution, and assigns them different weights by applying the attention mechanism.

**TABLE 1:** 5-CV performances of different prediction methods.

Methods	AUPR	AUC	F1	ACC	REC
AMMGC	0.239 9 ± 0.001 8	0.946 7 ± 0.000 7	0.318 4 ± 0.002 2	0.986 7 ± 0.000 8	0.358 7 ± 0.010 6
CF	0.204 6 ± 0.005 8	0.861 8 ± 0.005 8	0.287 3 ± 0.004 2	0.985 6 ± 0.000 7	0.331 4 ± 0.015 7
LP	0.226 2 ± 0.006 0	0.861 0 ± 0.003 9	0.307 5 ± 0.005 9	0.988 6 ± 0.001 0	0.317 6 ± 0.022 0
GF	0.161 9 ± 0.004 5	0.853 0 ± 0.002 7	0.231 8 ± 0.004 2	0.984 2 ± 0.001 0	0.274 5 ± 0.0170
SDNE	0.187 2 ± 0.007 4	0.869 3 ± 0.002 9	0.262 9 ± 0.007 4	0.985 3 ± 0.001 0	0.301 2 ± 0.017 7
GCMDR	0.224 2 ± 0.000 8	0.921 7 ± 0.000 2	0.304 9 ± 0.002 0	0.987 8 ± 0.000 6	0.309 8 ± 0.015 5

**FIGURE 2:**
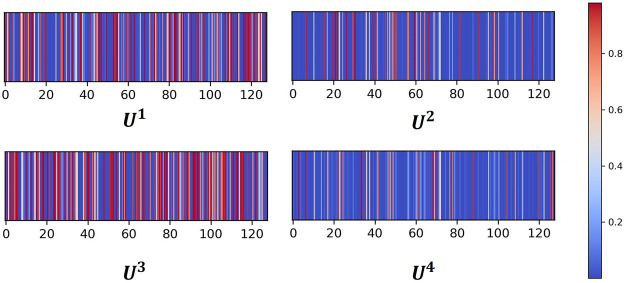
The heatmap of attention coefficients for four modals.

The attention neural network is one critical component of AMMGC, and it can measure and quantify the importance of each modal. Further, we visualize the attention coefficients for four modals in Figure 2. In general, four modals have different attention weights, and modal 1 and modal 3 have relatively higher weights than modal 2 and modal 4. This result shows AMMGC can well leverage different features to make predictions.

### 4.3 Ablation Analysis

In this section, we consider several variants of AMMGC to demonstrate the importance of the attention mechanism, multimodal and biological features. We provide a detailed analysis as follows.

• **AMMGC without attention network (w/o AN):** We assign the equal weights to all dimensions of multiple node embeddings from four graph convolution sub-networks.
• **AMMGC without biochemical feature (w/o BF):** Instead of using biological features of drugs and miRNAs, we simply employ one-hot encoding to build the graph convolution network.
• **AMMGC without multimodal (w/o MM):** Instead of using mutlimodal graph convolutional network, we directly concatenate two drug features and two miRNA features to build the prediction model.
• **AMMGC with modal 1 (w U1):** We train the AMMGC only with the modal 1.
• **AMMGC with modal 2 (w U2):** We train the AMMGC only with the modal 2.
• **AMMGC with modal 3 (w U3):** We train the AMMGC only with the modal 3.
• **AMMGC with modal 4 (w U4):** We train the AMMGC only with the modal 4.

The ablation results are shown in Table 2. The performance of AMMGC is much better than that of AMMGC without attention network in terms of all evaluation metrics, revealing that the attention neural network can exploit different contributions of embeddings for the prediction task and further improve the prediction performance. To verify the effectiveness of the graph convolution sub-networks, we compare AMMGC with AMMGC without Biofeature. Since biological features of drugs and miRNAs play vital roles in miRNA-drug resistance associations, the AUPR value drops by 4.17*%* and the REC value drops by 4.1*%* without them. More importantly, AMMGC performs better than AMMGC without Multimodal and the single-modal graph convolution models (AMMGC w U1/U2/U3/U4), indicating that the multimodal model can leverage diverse information to achieve better performances.

**TABLE 2:** Results of ablation study.

Models	AUPR	AUC	F1	ACC	REC
AMMGC	0.239 9 ± 0.001 8	0.946 7 ± 0.000 7	0.318 4 ± 0.002 2	0.986 7 ± 0.000 8	0.358 7 ± 0.010 6
AMMGC (w/o AN)	0.216 7 ± 0.002 3	0.944 6 ± 0.000 4	0.304 9 ± 0.004 4	0.986 3 ± 0.000 9	0.346 9 ± 0.018 4
AMMGC (w/o BF)	0.198 2 ± 0.000 5	0.941 4 ± 0.001 6	0.274 8 ± 0.002 0	0.985 4 ± 0.000 7	0.317 7 ± 0.015 7
AMMGC (w/o MM)	0.226 3 ± 0.012 9	0.944 5 ± 0.000 9	0.310 4 ± 0.008 8	0.985 5 ± 0.000 9	0.352 0 ± 0.007 0
AMMGC (w U1)	0.223 6 ± 0.002 8	0.944 2 ± 0.000 5	0.304 8 ± 0.002 8	0.986 2 ± 0.000 4	0.347 8 ± 0.010 1
AMMGC (w U2)	0.234 2 ± 0.001 4	0.942 4 ± 0.000 4	0.318 2 ± 0.003 1	0.986 4 ± 0.000 5	0.360 4 ± 0.013 2
AMMGC (w U3)	0.227 8 ± 0.002 4	0.944 3 ± 0.000 5	0.310 8 ± 0.002 9	0.986 6 ± 0.000 6	0.350 5 ± 0.017 9
AMMGC (w U4)	0.219 3 ± 0.002 0	0.940 9 ± 0.000 6	0.309 3 ± 0.003 5	0.986 0 ± 0.000 9	0.358 1 ± 0.015 7

### 4.4 Parameter Sensitivity Analysis

In this section, we investigate the sensitivity of parameters in AMMGC, including the number of graph convolution layers in sub-networks, the dimension of embeddings, and the negative sampling size.

**FIGURE 3:**
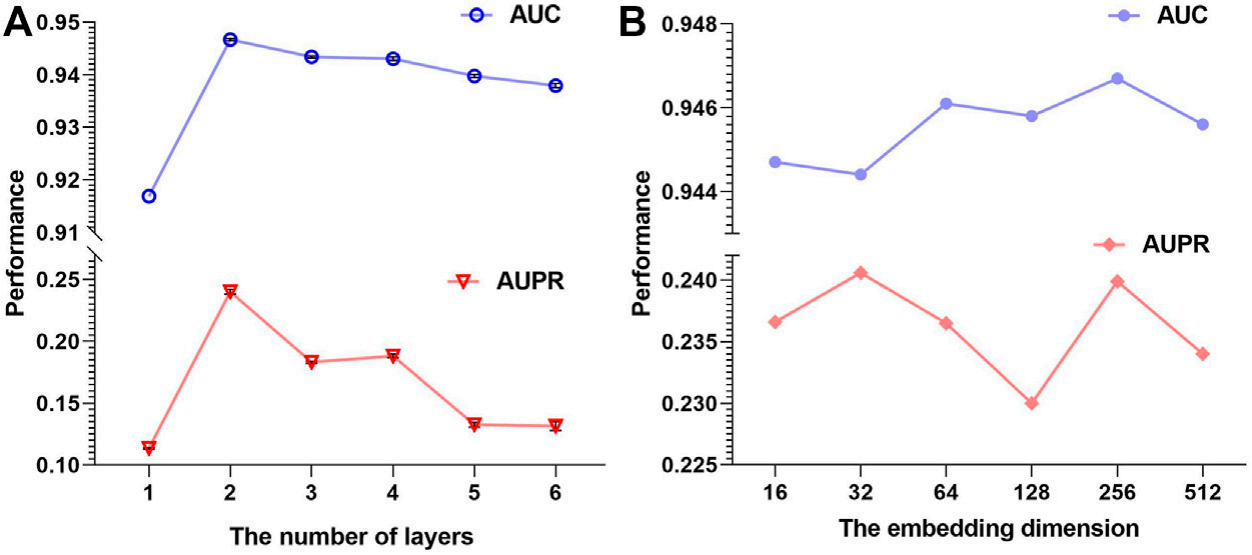
**(A)** AUPR and AUC of AMMGC with different GCN layers. **(B)** AUPR and AUC of AMMGC with different embedding dimensions.

For graph convolution sub-networks, we consider the number of layers from 1 to 6, and the results are shown in Figure 3A. The 2-layer GCN model achieves the best performance among all models, because 1-layer GCN may not learn sufficient information and more layers could lead to over-smoothing. In fact, related works have also shown that the two-layer GCN model can bring enough information and performs well (Li et al., 2019).

Embedding dimension is an important factor for our proposed method, which could directly influence the model performance. We fix the hidden units of the first GCN layer to 128 for all four graph convolution sub-networks and change the hidden units of the second GCN layer in {16, 32, 64, 128, 256, 512}. Thus, the final embedding dimension is in a range of {64, 128, 256, 512, 1024, 2048} according to Equation 4. From Figure 3B, we can see that different embedding dimensions lead to different performances. In general, the AUPR value varies around 0.235 and the AUC value varies around 0.946, which proves that our model performs stably if the embedding dimension is selected in an appropriate range. We finally choose the embedding dimension is 256 since the model with this setting has superior AUC and AUPR performance.

Since there are only positive samples in the dataset, negative samples are needed to conduct semi-supervised training on the prediction model. Thus, we randomly sample unlabeled miRNA-drug pairs to generate negative samples, and the size of negative sampling is fixed in each iteration. We conduct the experiments to evaluate the influence of the ratio *p* of the negative sampling size to that of the positive sampling size on the prediction performance. As shown in Table 3, AMMGC produces the greater scores on AUC, F1, and ACC with the increase of *p*, while producing the best AUPR and REC scores when *p* is set to 5. This result demonstrates the negative samples have an influence on the training of the AMMGC. Although the increase in the proportion of negative samples can bring slight performance improvements, the model takes more time to be convergent, so we choose to set *p* = 1 as the result of AMMGC.

**TABLE 3:** 5-CV Performances of AMMGC using different settings of negative sampling.

Value of p	AUPR	AUC	F1	ACC	REC
1	0.239 9 ± 0.001 8	0.946 7 ± 0.000 7	0.318 4 ± 0.002 2	0.986 7 ± 0.000 8	0.358 7 ± 0.010 6
5	0.241 5 ± 0.002 9	0.947 6 ± 0.000 9	0.319 6 ± 0.002 4	0.987 5 ± 0.000 6	0.357 3 ± 0.008 7
10	0.240 4 ± 0.003 6	0.948 1 ± 0.001 5	0.320 3 ± 0.002 7	0.988 1 ± 0.000 9	0.356 8 ± 0.008 1

### 4.5 Case Studies

In this section, we conduct case studies to test the capability of AMMGC in predicting novel miRNA-drug resistance associations. We train the AMMGC model with all known miRNA-drug resistance associations, then use the trained model to predict novel associations, which are further validated by public literature.

The results of case studies are shown in Table 4. According to Liao et al.’s research (Liao et al., 2015), hsa-mir-146a is found to influence the biologic features and prognosis of gastric cancer patients, and is associated with clinical characteristics in gastric cancer patients treated with adjuvant oxaliplatin and fluoropyrimidines. The association between gemcitabine and hsa-mir-145 is reported in Papadopoulos et al.’s work (Papadopoulos et al., 2015). In their study, hsa-mir-145 was significantly affected by gemcitabine treatment in T24 cells. Specifically, miR-145 levels were found to be dramatically upregulated (40.5-fold) during the first 36 h of treatment. In Hummel et al.’s research, they found Cisplatin can alter miRNA expression in esophageal cancer cells including hsa-mir-425 (Hummel et al., 2011). These studies show that AMMGC has the great potential of identifying novel miRNA-drug resistance associations.

**TABLE 4:** Top 10 miRNA-drug resistance associations predicted by AMMGC.

Drug	miRNA	Rank	Evidence
Gemcitabine	hsa-mir-30b	1	N.A.
Oxaliplatin	hsa-mir-146a	2	PMID: 26 396 533
Gemcitabine	hsa-mir-145	3	PMID: 25 833 690
Gemcitabine	hsa-mir-197	4	N.A.
doxorubicin	hsa-mir-363	5	N.A.
Gemcitabine	hsa-mir-320a	6	PMID: 23 799 850
5-Fluorouracil	hsa-mir-100	7	N.A.
Cisplatin	hsa-mir-425	8	PMID: 21 743 970
Gemcitabine	hsa-mir-23b	9	N.A.
Gemcitabine	hsa-let-7e	10	N.A.

## 5 Conclusion

In this work, we propose a deep learning-based method AMMGC to predict miRNA-drug resistance associations. AMMGC integrates multiple features of miRNAs and drugs to build graph convolution sub-networks, and learns node embeddings in different views. To obtain more comprehensive node embeddings, AMMGC employs an attention neural network to learn the contributions of different embeddings and assign them different weights for the final prediction. Experiment results demonstrate that integrating multiple features to build multimodal sub-networks is of vital importance for miRNA-drug resistance association prediction, and attention mechanism is also the key point to improve the performance.

There are several directions for our future study. On the one hand, AMMGC integrates side information about drugs and miRNAs under the graph convolutional network framework. More side information, such as interaction between drugs, miRNA-gene and gene-ontology relationship might be useful for predicting miRNA-drug resistance associations, and we hope to further investigate their usefulness. On the other hand, AMMGC is a general link prediction method, and it is promising to solve other related tasks.

## Data Availability

Publicly available datasets were analyzed in this study. This data can be found here: https://github.com/scz760904126/AMMGC.
